# Supplementary material for: MetaRibo-Seq measures translation in microbiomes
Source: Nat Commun. 2020 Jun 29;11:3268. doi: 10.1038/s41467-020-17081-z (PMC7324362; doi:10.1038/s41467-020-17081-z)
Supplement: Supplementary file 10 — Supplementary Data 7 [file 41467_2020_17081_MOESM10_ESM.zip › File2/Confidence_High_Taxonomy/200250_out.krona.html]

Javascript must be enabled to view this page.

members
magnitude
magnitudeUnassigned
count
unassigned
taxon
rank

200250\_out

12

2
12
superkingdom

201174
12
phylum

class
12
1760

12
85004
order

family
12
31953

12
1678
genus

8

SRS011239\_contig\_number\_17077SRS017103\_contig\_number\_26625SRS018351\_contig\_number\_9846SRS053356\_contig\_number\_33737SRS075878\_contig\_number\_13860SRS1041091\_contig\_number\_7409SRS140492\_contig\_number\_25619SRS146812\_contig\_number\_31900
1680
species

species
28026
4

SRS024331\_contig\_number\_25808SRS056273\_contig\_number\_11055SRS1055056\_contig\_number\_3802SRS147919\_contig\_number\_10954
